# Huwe1 supports B-cell development, B-cell-dependent immunity, somatic hypermutation and class switch recombination by regulating proliferation

**DOI:** 10.3389/fimmu.2022.986863

**Published:** 2023-01-09

**Authors:** Aldo Spanjaard, Maria Stratigopoulou, Daniël de Groot, Muhammad Aslam, Paul C. M. van den Berk, Chantal Stappenbelt, Matilda Ayidah, Joyce J. I. Catsman, Iris N. Pardieck, Maaike Kreft, Ramon Arens, Jeroen E. J. Guikema, Heinz Jacobs

**Affiliations:** ^1^ Division of Tumor Biology and Immunology, Netherlands Cancer Institute, Amsterdam, Netherlands; ^2^ Department of Pathology, Amsterdam University Medical Centers, Location Academic Medical Center (AMC), Lymphoma and Myeloma center Amsterdam (LYMMCARE), Amsterdam, Netherlands; ^3^ Department of Immunology, Leiden University Medical Center, Leiden, Netherlands

**Keywords:** HUWE1, B-cell, immune responses, homeostasis, development, somatic hypermutation, class switch recombination

## Abstract

The development and differentiation of B cells is intimately linked to cell proliferation and the generation of diverse immunoglobulin gene (*Ig*) repertoires. The ubiquitin E3 ligase HUWE1 controls proliferation, DNA damage responses, and DNA repair, including the base excision repair (BER) pathway. These processes are of crucial importance for B-cell development in the bone marrow, and the germinal center (GC) response, which results in the clonal expansion and differentiation of B cells expressing high affinity immunoglobulins. Here, we re-examined the role of HUWE1 in B-cell proliferation and *Ig* gene diversification, focusing on its involvement in somatic hypermutation (SHM) and class switch recombination (CSR). B-cell-specific deletion of *Huwe1* resulted in impaired development, differentiation and maturation of B cells in the bone marrow and peripheral lymphoid organs. HUWE1 deficiency diminished SHM and CSR by impairing B-cell proliferation and AID expression upon activation *in vitro* and *in vivo*, and was unrelated to the HUWE1-dependent regulation of the BER pathway. Interestingly, we found that HUWE1-deficient B cells showed increased mRNA expression of Myc target genes upon *in vitro* activation despite diminished proliferation. Our results confirm that the E3 ligase HUWE1 is an important contributor in coordinating the rapid transition of antigen naïve, resting B cells into antigen-activated B cells and regulates mutagenic processes in B cells by controlling AID expression and the post-transcriptional output of Myc target genes.

## 1 Introduction

The ability of B cells to protect the host from pathogens relies on their ability to terminally differentiate into plasma cells that secrete high-affinity antibodies in response to foreign antigen. During an immune reaction, B cells bind to and are activated by foreign antigen, resulting in the formation of a transient histological structure that include follicular dendritic cells and T follicular helper cells. Within the GC, B cells rapidly proliferate, and are licensed to undergo somatic hypermutation (SHM) to increase affinity of antibodies for target antigen (1, 2). Class switch recombination (CSR), which alters the effector function of antigen takes place in pre-GC B cells (3).

During SHM, mutations are introduced in the variable (V) regions of the immunoglobulin genes (*Ig*), which are instigated by activation-induced cytidine deaminase (AID). AID binds single-stranded DNA and processively deaminates cytosine (C) bases to uracils (U). Upon replication and if left unrepaired, the resulting U:G mismatches give rise to C-to-T and G-to-A transitions. Alternatively, the Us may be removed by the base excision repair (BER) pathway through the action of uracil glycosylase (UNG), yielding apurinic/apyrimidinic (AP) sites. AP sites can be bypassed by translesion synthesis (TLS) polymerases, generating C-to-T transitions, as well as C-to-A and C-to-G transversions. Conversely, the mutagenic fate of AP sites is counteracted by the AP endonucleases 1 (APE1) and APE2 that cleave the DNA phosphodiester backbone at the AP site, yielding a 5’ deoxyribose-phosphate (dRP) moiety and a 3’ hydroxyl group. The BER protein DNA polymerase beta (POLB) removes the 5’ dRP through its lyase activity and inserts C opposite G, thereby faithfully repairing the AID-instigated lesion. Lastly, the AID-instigated U:G mismatch may be recognized by the mismatch repair (MMR) components MSH2 and MSH6, which in concert with PMS2 and MLH1 recruit exonuclease 1 (EXO1). Subsequently, EXO1 excises a patch of DNA containing the U base(s) triggering fill-in synthesis by DNA polymerase eta (POLH), where POLH is recruited by PCNA-ubiquitination (4, 5). POLH is particularly error-prone when encountering A or T bases. This non-canonical MMR (ncMMR) activity results in A/T mutations in the V genes. Importantly, EXO1 activity requires a DNA nick as entry point to initiate excision. It was shown that PMS2 contains endonuclease activity that nicks 5’ from the U:G mismatch (6). Alternatively, the nick may be provided by the concerted action of UNG and APE1/2, suggesting that BER and ncMMR not only compete for the same U:G mismatch but also may cooperate to generate mutations (7).

During CSR the concerted action of AID, UNG, and AP endonucleases introduces staggered DNA double strand breaks (DSBs) at switch (S) regions located upstream of the constant regions within the *Igh* loci, enabling the deletion of gene fragments located between two active S regions in antibody Igh constant region (Ch), thereby exchanging Cμ (coding for IgM) for one of the downstream Ch regions. DSBs are generated when AP sites on opposite strands are sufficiently close and are cleaved by APE1/2. Alternatively, U:Gs are recognized as G mismatches by the MMR to generate DSBs by triggering DNA patch excision on both strands (8). As a result, the clonotypic class switched antibody of a B-cell is equipped with an alternate Ch with distinct effector functions and tissue distribution. Interestingly, the generation of DNA nicks required for CSR may be counteracted by conventional BER involving POLB, which faithfully repairs AID-instigated U lesions (9, 10).

Given these insights, it is apparent that both SHM and CSR involve the complex interplay between the BER and the MMR pathways, where the proximal part of the BER pathway (UNG and APE1/2) contributes to mutagenesis during SHM and to DNA nicks during CSR, whereas the distal BER component POLB counteracts mutagenesis and CSR. In agreement, we have recently shown that loss of POLB stimulates *in vitro* A/T mutagenesis by triggering EXO1 patch excision and ncMMR activity (10). Furthermore, we showed that human GC B cells are characterized by low protein expression of POLB, whereas B-cell lines that are used to study SHM and CSR *in vitro* display ample POLB protein expression, which may explain the relative paucity of A/T mutations in these cell lines.

The contribution of the BER and MMR pathways towards SHM and CSR has been extensively studied, however, the regulation of its components in a physiological context has received little attention. Previously, it was shown that the E3 ligase HUWE1 (also known as Mule or ARF-BP1) is an important modulator of the BER pathway, regulating the steady-state levels of POLB by ubiquitination (11). However, this was contested by Fang et al showing that lysine-to-arginine mutations of the HUWE1 target residues on POLB did not affect proteasome-mediated degradation (12). HUWE1 was shown to regulate additional DNA repair proteins by monoubiquitination, such as H2A.X histone variant (H2AX) and histone variant 2B (13, 14), breast cancer type 1 susceptibility protein (BRCA1) (15, 16), DNA polymerase lambda (POLL) (11, 17). In addition, HUWE1 was shown to be involved in DNA replication by interacting with proliferating cell nuclear antigen (PCNA) (13) and ubiquitination of cell division cycle 6 (CDC6) (18). Importantly, the proliferation-associated MYC proto-oncogene was shown to be a HUWE1 substrate. HUWE1-mediated lysine 63-linked (K63) polyubiquitination of MYC was required for recruitment of the transcriptional coactivator p300, thereby switching a repressive state of MYC into a transactivation state (19). MYC was shown to promote transcriptional elongation, which requires the interaction with polymerase II-associated factor 1 (PAF1c). Interestingly, the interaction between MYC and PAF1c is driven by HUWE1-mediated ubiquitination, showing that the transcriptional output of MYC is in part controlled by HUWE1 (14).

Previously, using B-cell-specific conditional *Huwe1*-deficient mice it was demonstrated that HUWE1 plays an important role in B-cell homeostasis (15, 20, 21) by preventing activation of the DNA damage response (DDR) involving the ataxia telangiectasia mutated (ATM) kinase and p53. It was shown that CSR *in vitro* and *in vivo* was impaired in Huwe1-deficient B cells. However, the GC response, the transcriptional consequences of *Huwe1* deletion in B cells and the effects on SHM were not reported (15). This prompted us to revisit the role of HUWE1 in B-cell activation and *Ig* diversification *in vivo*. In agreement with previous studies, we observed that HUWE1 was a crucial regulator of B-cell activation (15, 21). *Huwe1*;Mb1-cre mice harboured reduced B-cell numbers in the spleen and bone marrow. Stimulation of *Huwe1*;Mb1cre B cells failed to induce proliferation, despite increased upregulation of Myc target genes. In line with reduced proliferation, SHM, CSR, and GC formation were reduced in *Huwe1*;Mb1cre B cells. However, in contrast to previous reports we did not observe obvious effect of *Huwe1* deletion on POLB protein expression *in vivo*. Transcriptome analyses revealed that HUWE1 significantly impacted on the transcriptional output of Myc in resting and activated mature B cells. Our data highlight a critical contribution of *Huwe1* to B-cell homeostasis and *Ig* diversification mechanisms including CSR and SHM, by regulating proliferation and acting independently of the regulation of BER.

## 2 Materials and methods

### 2.1 Mice

*Huwe1*-floxed mice have been described elsewhere and were kindly provided by professor Wei Gu (Columbia University, Herbert Irving Comprehensive Cancer Center, New York, NY) (22). To obtain B-cell-specific deletions of *Huwe1*, Mb1-Cre homozygous male mice were crossed with *Huwe1*-FL/wildtype (WT) female mice. As HUWE1 is X-linked, this crossing generated male Mb1-Cre mice that were either *Huwe1*-WT or *Huwe1*-Floxed (Huwe1^FL/y^Mb1cre, indicated as *Huwe1*;Mb1cre in this study). The Mb1-Cre mouse model was chosen as it achieves efficient gene deletion in the B-cell lineage. Moreover, it allowed the recapitulation of previous findings on the role of Huwe1 in B-cell development and B-cell activation (15). For this study, 7 to 15-week-old mice were used. All animal experiments were approved by the Animal Ethics Committee of the Netherlands Cancer Institute (NKI) and the Dutch Experiments on Animals Act and the Council of Europe.

The genotyping primers to detect the WT, floxed, and deleted version of the *Huwe1* allele were 5’-CTAATCACAGGAAGCGGTTACAAG-3’, 5’-CTCCTATAGCAAAGTAAAAGTATAG-3’, 5’-GTTATTAGGTGGATCCGTACGAT-3’, and 5’-TTAGCTTGTTCTGCAGGTGGCGAC-3’. Primers for Mb1cre genotyping were 5’-CTGCGGGTAGAAGGGGGTC-3’ and 5’-CCTTGC

GAGGTCAGGGAGCC-3’. To assess *Huwe1* deletion in sorted B cells the following primers were used: 5’-GTGCAAGCTGAACAACAGGAA-3’ and 5’-AAGGAGAATGTGGATGCTGG-3’, which only yield a PCR product upon successful LoxP gene deletion. PCR settings were: 95°C (3min), 75°C (5min), 72°C (1.5min), followed by 40 cycles of 94°C (30sec), 58°C (45 sec), 72°C (45sec), and a final extension at 72°C.

### 2.2 Flow cytometry and FACS

To examine early B-cell development, bone marrow (BM) was flushed from the femurs using a 21-gauge syringe with cold PBEA buffer (1× PBS 0.5% BSA, 2 mM EDTA, and 0.02% sodium azide). Erylysis buffer (15.5mM NH_4_Cl, 1mM KHCO_3_, 0.01mM EDTA, pH 7.4) was added for 3min on ice to lyse red blood cells. Identical procedures were performed to obtain B cells from the spleen, except that here, spleens were first filtered through 70 µm strainers. Samples were measured using LSR Fortessa (BD Biosciences, Franklin Lakes, NJ) flow cytometers, and sorted using LSR Fortessa Aria2 cell sorter. Data was analyzed with FlowJo software v10.7.1 (Tree Star Inc., Ashland, OR).

### 2.3 Class switch recombination and proliferation

Spleens were isolated from 11-15 weeks-old mice. Single cell suspensions were generated and erylysis buffer was added to remove erythrocytes. Naïve splenic B cells were enriched by performing CD43 depletion using biotinylated anti-CD43 antibody (1:100, BD Pharmingen, San Diego, CA) and sorted from the spleen by flow cytometry-coupled sorting. 5 x 10^6^ B cells were labeled for 10 minutes at 37°C at a final concentration of 0,05 μM carboxyfluorescein succinimidyl ester (CFSE, Molecular probes, Eugene, OR) in Iscove’s Modified Dulbecco’s Medium (IMDM) containing 8% fetal bovine serum (FCS). For both the CSR and CFSE assays, the cells were seeded at 1 x 10^5^ cells per well. To trigger proliferation and CSR, the isolated B cells were stimulated with lipopolysaccharide (*Escherichia coli*, LPS, 5 μg/mL, Sigma-Aldrich, St. Louis, MO) and mouse recombinant interleukin-4 (IL-4) (10 ng/mL, Peprotech, London, UK), IL-4 and anti-CD40 (clone HM40-3, 1 μg/mL, BD Biosciences), and LPS combined with IL-4 and αIgD-dextran (1:5000, Fina Biosolutions LLC, Rockville, MD) to induce class switching. Cells were cultured for four days, at which the cells were harvested daily to measure proliferation. Additionally, after 72h and 96h, the percentage of cells that had class switched to IgG1 was measured.

### 2.4 Immunization and phenotyping the spleen

To analyze B-cell responses against infection, 7 – 11 weeks-old WT and *Huwe1;*Mb1cre mice were immunized by an intraperitoneal injection of 2 x 10^7^ lymphocytic choriomeningitis virus particles (LCMV-Armstrong, kindly gifted by Dr. Ramon Arens, Leiden). Two weeks after immunization, the spleens were isolated and single cell suspensions were made as previously described to investigate B-cell development (23). Splenocytes were labeled using the following markers for plasma cell identification (CD19, B220, IgD, IgM, Sca1, CD138, CD3e and Ter119), and for splenic mature B-cell subsets (CD19, B220, IgD, IgM, CD93, CD23and CD21/35) and GC staining (CD19, CD95, GL7 and IgM). Serum was prepared from blood to determine levels of LCMV-specific IgG and IgM by enzyme-linked immunosorbent assay (ELISA): In short, Nunc-Immuno Maxisorp plates (ThermoFisher Scientific, Waltham, MA) were coated overnight at 4°C with virus particles in bicarbonate buffer. Plates were subsequently incubated for 1h with blocking buffer (PBS/5% milk powder). Serum samples from mice were diluted in PBS/1% milk powder and incubated for 1h at 37°C. Next, Horseradish peroxidase (HRP)-conjugated IgG and IgM antibodies (Southern Biotech, Birmingham, AL) were diluted 1:4,000 in PBS/1% milk powder and incubated 1 h at 37°C. Plates were developed with 3,3’,5,5’-tetramethylbenzidine (TMB) substrate (Sigma Aldrich), and the color reaction was stopped by the addition of 1 M H_2_SO_4_. Optical density was read at 450 nm (OD_450_) using a Microplate reader (Model 680, Bio-Rad, Hercules, CA). Pre-immune serum samples were used as controls.

### 2.5 Somatic hypermutation analysis

To perform SHM analysis, splenic CD19^+^CD3^-^IgD^-^IgK^+^ memory B cells from unimmunized mice were obtained by FACS and the JH_4_ intron region was analyzed for mutations by DNA sequencing. This is a well-established and convenient method to analyze SHM in post-germinal center B cells (4). Briefly, memory B cells were sorted into tubes containing Tris-HCl and Proteinase K (ProtK). To lyse the cells, tubes were incubated for 1hr at 55°C, and ProtK was inactivated for 5 minutes at 85°C. Tubes were stored at -20°C until further use. DNA was purified by ethanol precipitation. The JH_4_ intron region was amplified by PCR using the VHB1-8 framework three primer forward primer (5’-CAGCCTGACATCTGAGGACTC-3’) and reverse primer (5’-CTCCACCAGACCTCTCTAGACA-3’). 2% E-gel with SYBR safe (Invitrogen) were used to extract the JH_4_ amplified product, corresponding to a fragment of around 650bp in length and sequenced by Illumina MiSeq Nano using paired-end sequencing. For mutation frequency calculations, identical mutated sequences were considered clonal and were counted once. Statistical analysis to determine the mutational spectra was performed in R to determine the mutational frequencies.

### 2.6 Protein extraction and Western blot

Spleens were isolated from 11-15 weeks-old unimmunized mice. Single cell suspensions were generated and erylysis buffer (hybridoma, red cell lysis buffer, Sigma-Aldrich) was added to remove erythrocytes. Naïve splenic B cells were enriched by performing magnetic-activated cell sorting (MACS) with anti-CD43 (Ly-48) MicroBeads, (miltenyi-Biotec, Bergisch Gladbach, Germany) according to the manufacturer’s protocol. The cells were seeded in in 24-well-plates (0.5 x 10^6^ cells per well) and activated with lipopolysaccharide (*Escherichia coli*, LPS, 50 μg/mL, Sigma-Aldrich) and IL-4 (20 ng/mL, ProspecBio, Hamada, Israel), αIgD-dextran (10ng/ml, Fina Biosolution) and recombinant human BAFF (100ng/ml, ProspecBio) for three days. After three days of activation the cells were harvested and washed with ice-cold PBS and lysed in ice-cold lysis buffer (10 mM Tris-HCl pH 8, 140 mM of NaCl, 1% Nonidet P-40, 0.1% sodium deoxycholate, 0.1% SDS, 1 mM of EDTA) supplemented with protease and phosphatase inhibitors (EDTA-free protease mixture inhibitor; Roche Diagnostics, Almere, the Netherlands). The protein pellets were homogenized passing through a 25G needle, and protein concentrations were measured using the bicinchoninic acid assay (BioRad). For each sample, 10 μg of total protein lysate and cell equivalents were used for protein separation in Precise 4–12% gradient Bis-Tris gels (Invitrogen, Waltham, MA). Subsequently, separated protein lysates were transferred onto polyvinylidene difluoride membranes (Immobilon-P; EMD Millipore, Burlington, MA); membranes were blocked in 5% BSA (BSA Fraction V; Roche Life Sciences, Almere, The Netherlands) in tris-buffered saline with 0.1% Tween-20 (TBS-T) for 1 h. Primary antibodies were incubated overnight at 4°C. After a series of thorough washes with TBS-T, membranes were incubated with secondary antibodies (goat anti-rabbit HRP or rabbit anti-mouse HRP; DAKO, Agilent Technologies, Heverlee, Belgium) for 2 h at room temperature. Antibody binding (protein expression) was visualized using Amersham ECL Prime Western blotting detection reagent (GE Healthcare Bio-Sciences AB, Uppsala, Sweden).

The following antibodies were used in this study: MSH6 (BD, 610919), MSH2 (clone FE11, Millipore), β-Actin antibody (clone AC-15, Sigma-Aldrich), DNA Polymerase beta antibody (ab26343, Abcam, Cambridge, UK), c-Myc (051203, Epitomics), p21 (SX118, Santa Cruz), AID [kind gift from Prof. Dr. Hans-Martin Jäck, University of Erlangen (24)].

### 2.7 Immunohistochemistry

Tissue sections were deparaffinized with xylene and hydrated through an alcohol rinse series followed by 15 minutes incubation in methanol + 0.3% H_2_O_2_. After heat-inducible epitope retrieval in citrate buffer (pH=6) in a pressure cooker for 20 minutes, slides were treated with Super Block (AAA125, ScyTek Laboratories, West Logan, UT) for 15 minutes at room temperature. Then, the tissue sections were incubated with the primary antibodies (PNA, Vector laboratories, B-1075; POLB, Abcam, ab26343, POLB antibody specificity was confirmed by immunohistochemistry on paraffin-embedded CH12-F3 Polb-knockdown mouse lymphoma cells that were described previously (10); Ki-67, ThermoFisher scientific, SP6). Antibody–antigen detection was performed with goat anti-rabbit poly horseradish peroxidase (HRP) (Bright Vision poly-HRP-anti-rabbit IgG, Immunologic, VWR, Duiven, The Netherlands). The end products were visualized with 3,3’-diaminobenzidine (DAB) (Bright DAB substrate kit, Immunologic). For double immunohistochemistry stainings, similar to single staining, tissue sections were deparaffinized. After heat induced-epitope retrieval and blocking with Super Block, the cells were stained for c-Myc (051203, Epitomics) overnight at 4°C. After several washing, slides were incubated with secondary goat-anti-rabbit-poly-alkaline phosphatase (AP) (Bright Vision poly-AP-anti-rabbit IgG, Immunologic) for 30 minutes at room temperature, followed by adding VECTOR® Red AP substrate, which resulted in a red precipitate. The slides were then boiled in citrate buffer (pH=6) again to remove the bound antibodies, follow by blocking with Super Block and restained with rat anti-p21(HUGO291, Abcam) overnight at 4°C. After several washing steps, slides were incubated with secondary rabbit-anti-rat antibodies (DAKO) for 30 minutes at room temperature and subsequently with goat anti-rabbit-poly-AP (Bright Vision poly AP-anti-rabbit IgG, Immunologic) for 30 minutes at room temperature followed by adding VECTOR® Blue AP substrate, resulting in blue precipitate. The slides were covered with VECTAMOUNT® permanent mounting medium (H-5000, Vector laboratories, Burlingame, CA).

### 2.8 Statistics

Statistical analyses were performed using Graphpad V7.0. Statistical significance was calculated using an unpaired student *t-*test with Welch correction to account for unequal standard deviations. For the somatic hypermutation analysis, Fisher’s exact chi-square test was performed.

## 3 Results

### 3.1 Huwe1 controls B-cell development in the bone marrow and spleen

**Figure 1:**
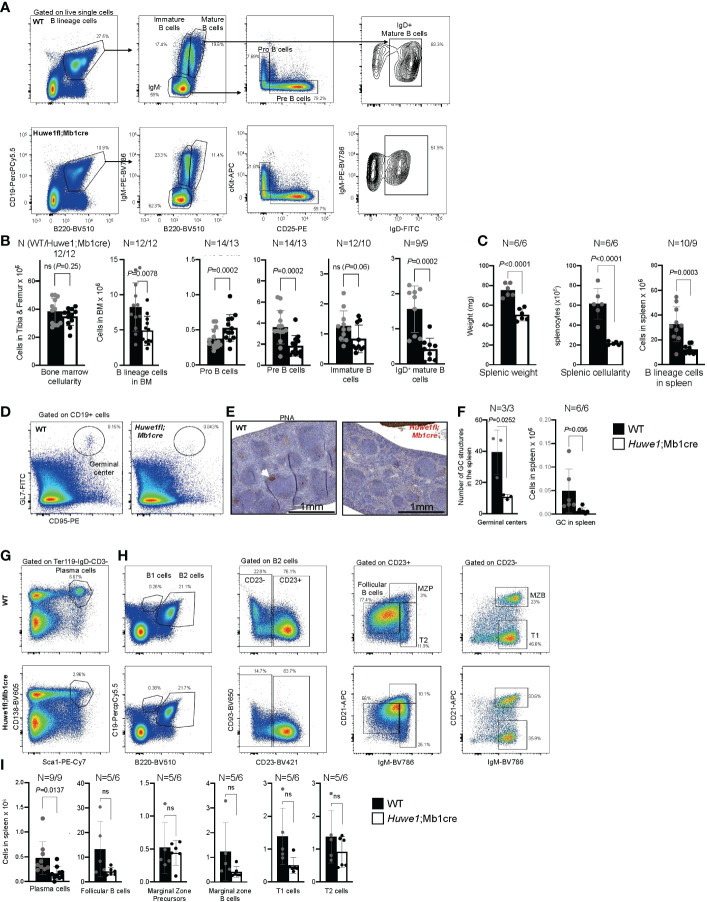
Impaired B-cell development in the bone marrow of *Huwe1*;Mb1cre mice. **(A)** Representative flow cytometry gating strategy to assess B-cell development in the bone marrow of wildtype (WT) mice (top) and *Huwe1*;Mb1cre (bottom) mice. Left panels show the B-cell percentages in the nucleated cell compartment, middle panels show the IgM-negative, mature, and immature B cells, and the right panels depict the pro-B cells and pre-B cells. **(B)** Absolute numbers of cell populations indicated in **(A)** in the bone marrow. Mean ± SD is shown. N for WT (left) and *Huwe1*;Mb1cre is depicted above the graph. Black bars depict WT mice, white bars depict *Huwe1*;Mb1cre mice. **(C)** Splenic weight, cellularity, and B lineage cells (CD19^+^ B220^+^) in the spleen. **(D)** Flow cytometry gating strategy of spontaneous germinal centers in the spleen. Percentages of GL7+ CD95+ GC B cells are indicated. **(E)** Peanut agglutinin (PNA) immunohistochemistry staining in the spleen. Magnification is 20X. **(F)** Germinal centers in the spleen as identified by counting PNA+ GCs in IHC slides (left), and by flow cytometry staining for GL7 and CD95 (right). **(G, H)** Flow cytometry gating strategy for plasma cells in the spleen and splenic follicular B cells, marginal zone progenitors (MZP) and transitional 1 (T1) and 2 (T2) B-cell subsets **(H)**. **(I)** Absolute numbers of subsets as indicated in **(G, H)**, bar graphs depict means ± SD. *P*-values were calculated using an unpaired t-test with Welch correction. ns, not significant.

To re-examine the effects of HUWE1 deficiency in murine B cells, we crossed male homozygous *Mb1cre* mice with female *Huwe1fl/wt* mice from a strain (22) that is different than the mouse strain for which B-cell specific defects were reported previously (15). Resulting male offspring featured either a WT or floxed version of *Huwe1* (indicated as *Huwe1*/Mb1cre), which could be detected *via* PCR. We could also identify deletions of *Huwe1 via* PCR in sorted splenic B cells (**Supplementary Figures 1A-E**). Comprehensive analysis of B-cell development in the bone marrow of *Huwe1*;Mb1cre mice confirmed a decrease of B-lineage cells in the bone marrow (**Figures 1A, B**). While the cellularity of the bone marrow was not altered in *Huwe1*;Mb1cre mice (**Figure 1B**), B cells were reduced in number (**Figure 1B**). Pro-B cells were increased in numbers, in contrast to what has previously been reported (15) and pre-B cells were significantly reduced in *Huwe1*;Mb1cre mice (**Figure 1B**), suggestive of a pro- to pre-B cell differentiation block. We did not observe significant differences in the number of immature B cells (**Figure 1B**). While in WT mice, we could clearly distinguish immature from mature B cells on the basis of B220 staining, this discrimination was less apparent in *Huwe1*;Mb1cre mice due to reduced expression of B220 (**Figure 1A**). We therefore gated on IgD-positive B220-positive mature B cells and found that their numbers were significantly decreased in *Huwe1*;Mb1cre mice (**Figures 1A, B**).

The effects of *Huwe1*-deficiency were even more pronounced in the spleen. We noted a significant decrease in splenic weight, splenic cellularity, and splenic B-cell numbers in absence of Huwe1 (**Figures 1D–I**). Moreover, fewer plasma cells and spontaneous GCs were observed in the spleens from unimmunized mice (**Figures 1D–F**), as shown by both flow cytometry and by immunohistochemical staining with the peanut agglutinin (PNA) (**Figures 1D–F**). Consistent with the reduction of GC B cells as defined by flow cytometry, we observed less and far smaller spontaneous GC structures in the spleens of unimmunized animals (**Figure 1F**). These results are in line with prior observations of *Huwe1*;Mb1cre mice, and firmly establish the role of Huwe1 in B-cell development in the bone marrow and spleen (15, 20, 21). However, while we observed a decrease of multiple types of B-cell subsets, such as B1a, B2, T1, T2, cells *etc.*, except for plasma cells, none of these subsets was significantly decreased, suggesting that differentiation of B cells to differentiated subsets is not affected significantly by the absence of *Huwe1*.

### 3.2 Reduced but not abolished immune responses in *Huwe1*-deficient B cells

**Figure 2:**
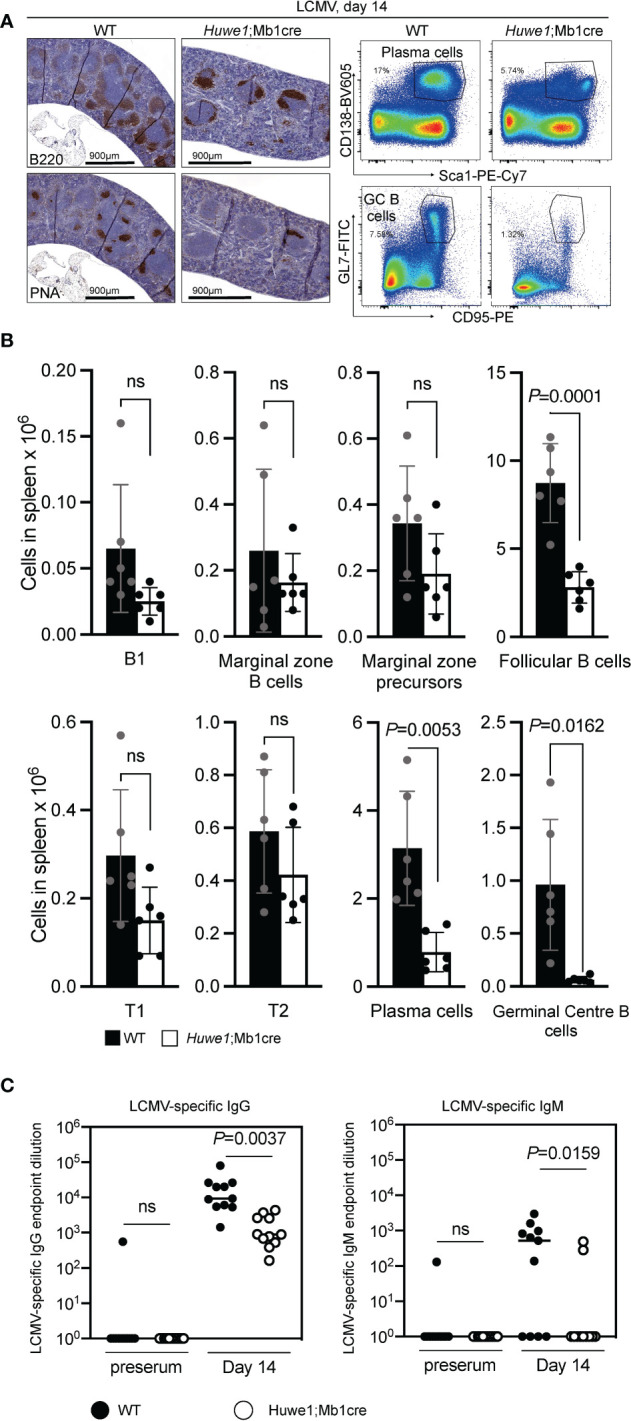
Huwe1-deficiency reduces the immune response to lymphocytic choriomeningitis virus. (LCMV). Wildtype (WT) and Huwe1;Mb1cre mice were infected with LCMV and analyzed 14 days post-infection. **(A)** Left panels show immunohistochemistry for B220 (total B cells) and peanut-agglutinin (PNA) (GCs). Right panels show flow cytometry gating strategy for plasma cells (CD138+ Sca1+) and GC B cells (GL7+ CD95+) in spleens from LCMV-infected mice. Percentage of gated cells are indicated. **(B)** Absolute numbers of indicated B-cell subsets from LCMV-infected spleens. Means ± SD are depicted. Black bars represent WT mice, white bars represent Huwe1;Mb1cre mice (N=6 for both groups). Flow cytometry gating strategy for B-cell subsets was similar as in Figure 1. **(C)** LCMV-specific IgG and IgM serum titres 14 days post-infection. Serum isolated before infection (preserum) was used as control. Numbers indicate the endpoint dilution of the sera. N=12 for both groups. P-values were calculated using an unpaired t-test with Welch correction. ns, not significant.

To assess whether GC responses were also affected in immunized *Huwe1*;Mb1cre mice we examined immune responses to lymphocytic choriomeningitis (LCMV)-virus, which elicit robust and relevant T-cell dependent B-cell responses that includes GC reaction, memory B-cell and long-lived plasma cell formation. At day 14 post LCMV-infection, spleens and serum samples were collected for analysis. Immunohistochemistry revealed reduced levels of B cells and GCs in the spleen after LCMV infection, which was confirmed using flow cytometry (**Figures 2A, B**; see (23) for extended gating strategy). In accordance, splenic plasma cells were decreased in *Huwe1*;Mb1cre mice (**Figure 2B**). In parallel, LCMV-specific IgM and IgG serum titres were determined. We noted a significant decrease in serum levels of LCMV-specific IgG and IgM antibodies (**Figure 2C**), the decrease being most prominent for IgM titers. The LCMV-specific IgG response was approximately 10-fold reduced in the *Huwe1*;Mb1cre mice, but not ablated, suggesting that a GC-independent humoral response, perhaps involving the differentiation of marginal zone (MZ) B cells, may support the LCMV-specific IgG in *Huwe1*;Mb1cre mice. Importantly, loss of HUWE1 did not significantly affect differentiation of splenic B cells, although there was a trend that B-cell levels were reduced (**Figure 2B**). However, these results suggest that while development of the B lineage cells relies on HUWE1, once B-cell identity has been determined, much of the downstream differentiation is not significantly affected, with the exception for GC B cells and plasma cells.

### 3.3 HUWE1 is essential for *in vitro* B-cell proliferation and CSR and affects SHM *in vivo*

**Figure 3:**
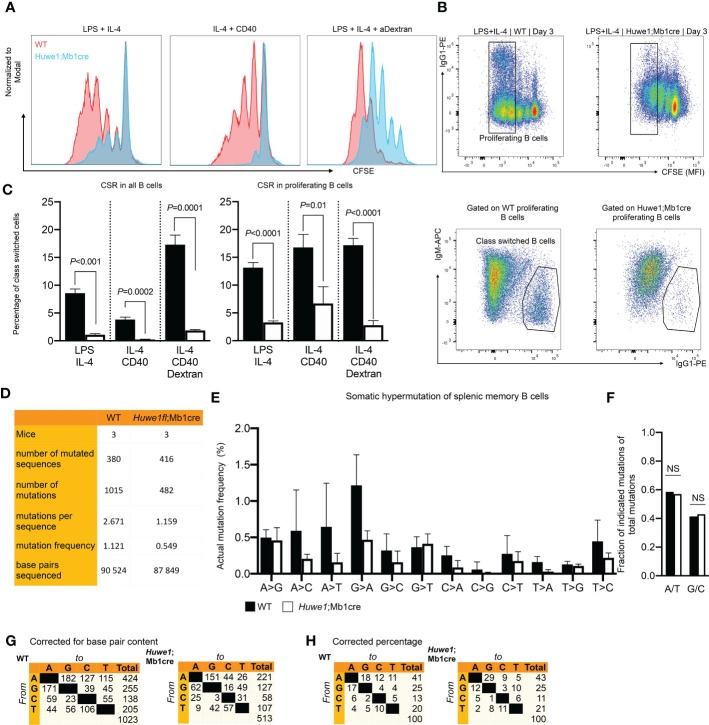
Reduced proliferation, class switch recombination (CSR), and somatic hypermutation. (SHM) in *Huwe1*;Mb1cre mice. **(A** Proliferation of activated B cells as measured by carboxyfluoroscein succinimidyl ester (CFSE) labeling. Resting splenic B-cells were activated *in vitro* for 3 days as indicated and analyzed by flow cytometry. CFSE overlay histograms are shown. Splenic B cells from wildtype (WT) mice are depicted as red histograms, B-cells from *Huwe1*;Mb1cre are shown as blue histograms. One of three representative experiments is shown. **(B)** Splenic B-cells activated for 3 days with lipopolysaccharide (LPS) and interleukin-4 (IL-4) to induce IgG1 CSR. Activated B cells were analyzed by flow cytometry. CSR was determined by staining for IgG1. **(C)** Percentages of IgM- IgG1+ cells in all activated B cells (left panel, no gate applied on CFSE channel) and in proliferating B cells (right panel: gating strategy shown in B, designated as ‘proliferating B cells’). Bar graphs depict means ± SD, one representative of three independent experiments is shown. **(D)** SHM was determined in splenic memory B cells (CD19+ CD3- IgD- IgK+) isolated from unimmunized mice. SHM was assessed by next-generation sequencing of JH4 intron amplicons. Table indicates numbers of mice, sequences and mutations analyzed. **(E)** Frequencies of mutations, defined as number of specific template base pairs to be sequenced to find the indicated mutation in WT and *Huwe1*;Mb1cre B-cells. Means ± SD of three mice are depicted. **(F)** Bar graph depicting frequencies of mutations at A and T bases (A/T), or G and C bases (G/C) (no significant differences as determined by Fisher’s exact chi-square test). **(G-H)** Cross tables showing the mutation spectrum, indicated as total number of mutations **(G)** or the percentage of total mutations **(H)**. *P*-values were calculated using an unpaired t-test with Welch correction. ns, not significant.

The reduced B-cell development in bone marrow and spleen, and the blunted GC responses in absence of Huwe1 could result from defects in proliferation, differentiation, or increased apoptotic signalling. To determine the effects of Huwe1 deficiency on the proliferative capacity of B cells we activated resting splenic B cells for three days with various B-cell stimulation cocktails that induce CSR to IgG1, as CSR is is intimately linked to B-cell proliferation (25). Proliferation of *Huwe1*;Mb1cre B cells was decreased compared to WT B cells after stimulation with LPS and IL-4, IL-4 and CD40, and LPS and IL-4 and anti IgD-dextran as shown by CFSE dilution analysis (**Figure 3A**). Concomitantly, IgG1 CSR was severely impaired in all stimulations tested (**Figures 3B, C**). Since CSR requires cell division, and as *Huwe1*;Mb1cre B cells featured less cell division after stimulations, we analyzed the level of CSR in equally divided B cells (**Figure 3C**). Of interest, even in B cells that underwent an equal number of cell divisions, *Huwe1*-deficiency impaired CSR, indicating that *Huwe1* controls CSR in a proliferation-independent manner.

To assess whether the defective GC response also affected SHM we isolated Ig-Kappa-positive, IgD-negative splenic memory B cells from unimmunized mice, and used JH4-intron next generation sequencing to determine SHM. To circumvent any potential bias due to fewer GCs found in *Huwe1*;Mb1cre mice we chose to analyze SHM in positively selected post-GC (memory) B cells. We noted a strong decrease in the mutation load of SHM affecting nearly the entire spectrum of mutations, indicating that absence of HUWE1 does not affect the generations specific classes of mutations (transversions and transitions at specific bases, **Figures 3D–G**). In line with this notion, there was no significant difference (Fisher’s exact chi-square, p=0.6) in the frequency of mutations at A:T and C:G bases (**Figure 3F**). These results argue against any direct role of HUWE1 in SHM with regards to regulating DNA repair proteins implicated in the SHM mutation spectrum (7), but rather argue that lower mutation loads of HUWE1-deficient B cells is the result of reduced proliferation and/or lower AID expression.

### 3.4 HUWE1 regulates the transcriptional output of MYC in B cells

**Figure 4:**
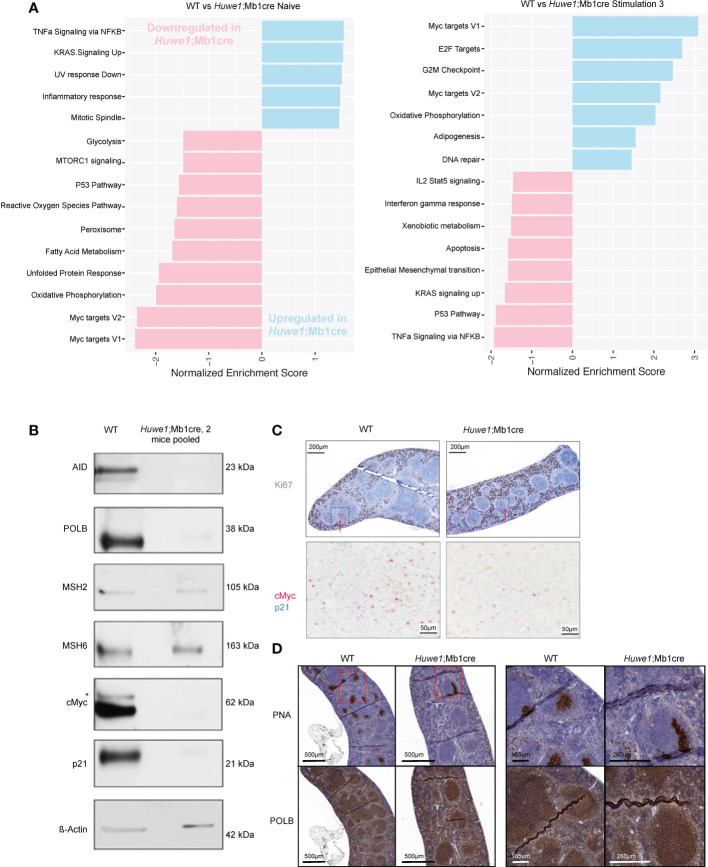
RNA-sequencing gene expression profiling and protein expression analysis of HUWE1-deficient B cells. **(A)** Fast gene set enrichment analysis (FGSEA) of naive and stimulated B cells. Plotted is the normalized enrichment score (NES) of genes down and upregulated in *Huwe1*;Mb1cre (pink and blue, respectively.) For each condition, RNA-sequencing was performed on B cells from three independent mice. **(B)** Immunoblotting analysis of protein expression in activated splenic B cells stimulated with LPS+IL4+anti-IgD-dextran (‘Stimulation type 3’). For each sample, 10 μg of total protein were used for protein separation. To obtain sufficient material, B cells from 2 *Huwe1*;Mb1cre mice were pooled. Aspecific band in the cMyc immunoblot is indicated with an asterisk. β-actin is used as a loading control. **(C)** Immunohistochemistry (IHC) staining for Ki67 (visualized by 3,3’-diaminobenzidine substrate, brown color) to identify proliferating cells and germinal centers in formalin-fixed paraffin-embedded spleen sections from LCMV-infected wildtype (WT) and *Huwe1*;Mb1cre mice 14 days post-infection. The red arrows indicate the GC structures. The area surrounded by the box with the black dashed line corresponds to the area shown in the bottom panel of the figure studying by IHC the cells that express cMyc (pink) and p21 (blue) in splenic GCs from WT and *Huwe1*;Mb1cre unimmunized mice. **(D)** IHC staining for peanut agglutinin (PNA) to identify GCs, and DNA polymerase beta (POLB) in the spleens from LCMV-infected mice 14 days post-infection. Panels on the right are magnifications of the indicated boxes with the red dashed lines.

To further investigate these effects of Huwe1 deficiency we examined the transcriptomes of WT and *Huwe1*;Mb1cre B cells by RNA-seq. We isolated resting B cells from the spleens of unimmunized mice, and either directly isolated RNA, or stimulated B cells for three days with LPS and IL-4 and anti-IgD-dextran. Importantly, upon stimulation, we noted decreased expression of *Huwe1* mRNA in *Huwe1*;Mb1cre cells, indicating efficient deletion of *Huwe1*. Moreover, stimulated *Huwe1*;Mb1cre B cells featured reduced mRNA expression of *Aicda*, encoding AID, which partly explains the impaired CSR in stimulated *Huwe1*;Mb1cre B cells (**Supplementary Figure 2A**). Gene set enrichment analysis (GSEA) revealed that in unstimulated B cells, HUWE1-deficiency resulted in lower expression of MYC target genes and p53 pathway genes, in line with the reported role of HUWE1 on Myc (20, 21). In stimulated B cells, however, we noted significantly increased mRNA expression of the Myc target genes, as well as DNA repair genes, while p53 pathway genes remained downregulated upon stimulation in *Huwe1*-deficient B cells compared to WT B cells (**Figure 4A**). Strikingly, the genes that contributed most significantly to the MYC gene set enrichment (‘leading-edge genes’) in unstimulated WT B cells largely overlapped with the leading-edge genes responsible for MYC gene set enrichment in stimulated *Huwe1*-deficient B cells (**Supplementary Figures 3**, **4**), suggesting that HUWE1 maintains the promoters of this set of genes in a poised state in unstimulated B cells and is involved in the post-transcriptional control of these genes in stimulated B cells.

### 3.5 HUWE1-deficiency does not result in POLB stabilization in activated B cells

In addition, we analyzed the expression of relevant proteins by comparative immunoblotting of lysates obtained from LPS and IL-4 and anti-IgD-dextran-stimulated WT and *Huwe1*;Mb1cre B cells. In agreement with the transcriptome data, activated *Huwe1*;Mb1cre splenic B cells showed reduced protein expression levels of AID (**Figure 4B**). Opposite of what we expected, the expression of POLB was decreased, indicating that loss of HUWE1 did not result in POLB protein stabilization (**Figure 4B**), as was previously suggested (11). Strikingly, *Polb* mRNA expression was not affected in activated *Huwe1*;Mb1cre B cells, suggesting a post-translational regulation of POLB that did not involve HUWE1-dependent ubiquitination and proteasomal degradation. Of interest, other proteins associated with (error-prone) processing of AID-instigated lesions, such as the mismatch repair proteins MSH2, MSH6 were not affected by HUWE1 deficiency, which showed that lower expression of DNA repair proteins is not a general feature of activated *Huwe1*;Mb1cre B cells. While the mRNA transcription of Myc target genes was significantly enriched, the Myc protein level was reduced in activated *Huwe1*;Mb1cre cells compared to WT cells, as was the level of cell cycle regulator p21, which is a direct transcriptional target of p53 (**Figure 4B**). We studied the expression of Myc and p21 also *in situ* and we confirmed their downregulation in the splenic GCs of LCMV-infected *Huwe1*;Mb1cre mice at day 14 compared to WT mice (**Figure 4C**). POLB was expressed in splenic GCs from LCMV-immunized mice, in stark contrast to continual GC in human tonsils, which we reported previously to be characterized by low POLB expression (10). Specificity of the POLB antibody used for immunohistochemistry was confirmed by immunoblotting and immunohistochemistry on paraffin-embedded CH12/F3 Polb knockdown mouse GC-derived lymphoma cells (**Supplementary Figures 5A, B**). Although POLB protein expression was decreased in *in vitro* activated *Huwe1*;Mb1cre B cells, expression in GCs *in situ* was not affected (**Figure 4D**).

Our results show that HUWE1 crucially regulates B-cell proliferation, which impinges on B-cell progenitor expansion in the bone marrow, splenic B-cell numbers, CSR and SHM. Our data are not consistent with a role for HUWE1 in the regulation of POLB or other DNA repair proteins that may affect CSR and SHM. However, HUWE1 appears to participate in the control of MYC-target genes. Our finding that the mRNA expression of many DNA replication-associated genes was consistently upregulated in activated *Huwe1*/Mb1cre B cells, although these cells were severely hampered with regards to proliferation, suggests that HUWE1 may play a crucial role in the post-transcriptional/post-translational regulation of the products of these genes.

## 4 Discussion

In this study we aimed to delineate the relevance of the E3 ligase HUWE1, also known as Mule or ARF-BP1 in B-cell-dependent immunity. *Huwe1* is essential for life, as a bi-allelic inactivation was found to be embryonic lethal (15, 22). However, consistent with a previous study (15), B-lineage cells are able to tolerate partially the loss of Huwe1 at distinct stages of B-cell development. An ablation of both *Huwe1* alleles early in the B-cell lineage caused a developmental block of B-cell progenitors at the transition from pro- to pre-B cells. Yet, immature and mature B cells do develop and are present in the peripheral blood and in the spleen, albeit at reduced numbers, indicating that development is hampered but not prohibited by the loss of *Huwe1* expression. These insights argue against a critical role of HUWE1 in B-cell development and differentiation, but rather highlights an important contribution of the regulation of cell proliferation.

Given the relevance of HUWE1 in controlling central processes like cell proliferation and DNA-repair (11, 13, 14), we wanted to determine to what extent this affects the intimate link between GC B-cell proliferation and secondary *Ig* gene diversification, two important hallmarks of the GC reaction. In line with a previous report on the role of HUWE1 in B-cell functioning, proliferation and differentiation of B cells were hampered in the absence of HUWE1 (15). Moreover, detailed *ex vivo*, as well as *in vitro* CSR and SHM analyses combined with transcriptomics identify HUWE1 as an important driver of B-cell activation, and in extension, the GC reaction. In agreement with the failure of *Huwe1-*deficient B cells to upregulate AID expression at the transcriptional and translational level, activated B cells were grossly impaired in their CSR activity *in vitro*, as well as their SHM capacity *in vivo*. The failure to acquire normal AID expression levels in the absence of *Huwe1* explained the global reduction of the mutation load in somatically mutated *Ig* genes and the decreased CSR activity. We hypothesize that reduced proliferation resulted in lower AID expression, which is linked to cell division. We observed fewer GCs in *Huwe1*;Mb1cre mice, and consequently, fewer output cells (memory B cells and plasma cells). SHM was generally lower in positively selected post-GC memory B cells without affecting the mutation spectrum, consistent with lower AID levels during the GC reaction in *Huwe1*;Mb1cre mice.

Interestingly, it was shown that Myc overexpression was able to complement the CSR defect in Huwe1-deficient B cells (21). However, Myc has not been reported to directly drive AID transcription in GC B cells. This suggests that the effect of Huwe1 deficiency on AID expression indirectly involved Myc, most likely by diminished Myc output resulting in hampered proliferation, thereby limiting AID expression.

In the hematopoietic stem cell (HSC) compartment, *Huwe1* controls lineage differentiation, stress responses, and stem cell gene expression (20). The strong reduction of B cells in *Huwe1*;Mb1cre mice suggested HUWE1-dependent deregulation of pathways crucial for B-cell development and lineage choice. Analyses of the transcriptomes of WT and *Huwe1*-deficient B cells showed however, that very few pathways were significantly differentially expressed. The most significantly affected genes belonged to the MYC_v1/MYC_v2 gene sets, whose expression was downregulated in naïve (unstimulated) *Huwe1*-deficient B cells compared to WT B cells, as expected. Counterintuitively, most of these genes were found to be upregulated in activated (LPS+IL-4+anti-IgD-dextran stimulated) *Huwe1*-deficient B cells compared to activated WT B cells. Many of these genes are important for cell-cycle progression, such as *CDK4*, *MCM2*, *MCM6*, *PCNA* and *POLD2*, yet the *Huwe1*-deficient B cells showed severely hampered *in vitro* proliferation upon activation. Moreover, MYC protein expression was clearly lower in activated *Huwe1*;Mb1cre B cells, arguing that even in the absence of MYC, gene expression of MYC target genes can occur, although this clearly does not promote efficient replication. Various studies suggest that HUWE1 critically regulates MYC family members in a context-dependent manner (20, 21). For instance, in HSCs it was demonstrated that N-myc was upregulated upon loss of Huwe1 leading to increased proliferation and stem cell exhaustion, which was reversed by *Mycn* depletion (20). Whereas in B cells it was shown that the homeostatic defects caused by loss of Huwe1 could be mitigated by overexpression of C-myc (21). Based on this we speculate that in the absence of HUWE1, proliferation of B cells may be initiated but cannot proceed normally due to the absence of functional MYC. This may be accompanied by the inappropriate mRNA expression of DNA replication-associated genes due to defective cell-cycle progression and cell-cycle regulated transcription. Alternatively, but not mutually exclusive, HUWE1 may be involved in the post-transcriptional/post-translational regulation of these genes, by which increased mRNA expression is not effectuated in enhanced function of the gene products. Also, our results suggest that the increased expression of MYC target genes is not necessarily related to increased MYC protein expression, indicating that other transcription factors may crucially regulate the mRNA expression of MYC target genes in the absence of MYC protein. To further elucidate this mechanism additional molecular investigations are required.

A previous study in human cell lines identified DNA Polymerase Beta (POLB), which exerts a critical function in short patch BER, as a target of the E3 ligase HUWE1 (11). However, in contrast to an expected stabilization of POLB, POLB production was reduced in activated murine *Huwe1-*deficient B cells, perhaps due to differential proliferation. Moreover, in stark contrast to human GCs, levels of POLB were not found to be reduced in murine GCs compared to the surrounding tissue. Hence, in the murine setting, the lack of *Huwe1* could not be associated with a stabilization of POLB, neither *in vitro* nor *in situ*. The decreased level of POLB in activated B cells could moreover be explained by the decreased levels of p53 since there is evidence of p53 implication in the BER and most specifically POLB *in vivo* (26). At the same time, members of the MMR machinery, specifically the mismatch recognition complex MSH2/6 were found unaffected *in vitro*. These insights are consistent with the notion that despite an overall reduction in the SHM load, the spectrum of SHM was found remarkably stable between both conditions, having no impact on A:T mutations versus G:C mutations.

Our findings identify HUWE1 as a critical regulator of B-cell mediated immunity. In addition to proliferation, HUWE1 regulates critical pathways associated with B-cell activation, SHM, and CSR. Our study demonstrates the complexity of Huwe-1 in controlling cell proliferation. To distinguish from direct and indirect effects, future studies will have to identify direct and indirect targets of HUWE1 and define their spatio-temporal relation during the cell-cycle and define the targets steering B-cell development and differentiation.

## Data availability statement

RNA sequencing data has been deposited to the NCBI GEO database and can be accessed under accession number GSE221351. The somatic hypermutation NGS dataset has been deposited at the NCBI SRA database and can be accessed under accession number PRJNA913839.

## Ethics statement

All animal experiments were approved by the Animal Ethics Committee of the Netherlands Cancer Institute (NKI) and the Dutch Experiments on Animals Act and the Council of Europe.

## Author contributions

AS, PB, MuA, JG and HJ designed the study. AS, MS, JG and HJ wrote the manuscript. AS, MS, CS, MA, JC MK, and IP performed wet-lab experiments. DG and MuA analyzed the transcriptome data and performed the somatic hypermutation analysis. All authors contributed to the article and approved the submitted version.
