# Supplementary figures and images for: Huwe1 supports B-cell development, B-cell-dependent immunity, somatic hypermutation and class switch recombination by regulating proliferation
Source: Front Immunol. 2023 Jan 9;13:986863. doi: 10.3389/fimmu.2022.986863 (PMC9869049; doi:10.3389/fimmu.2022.986863)

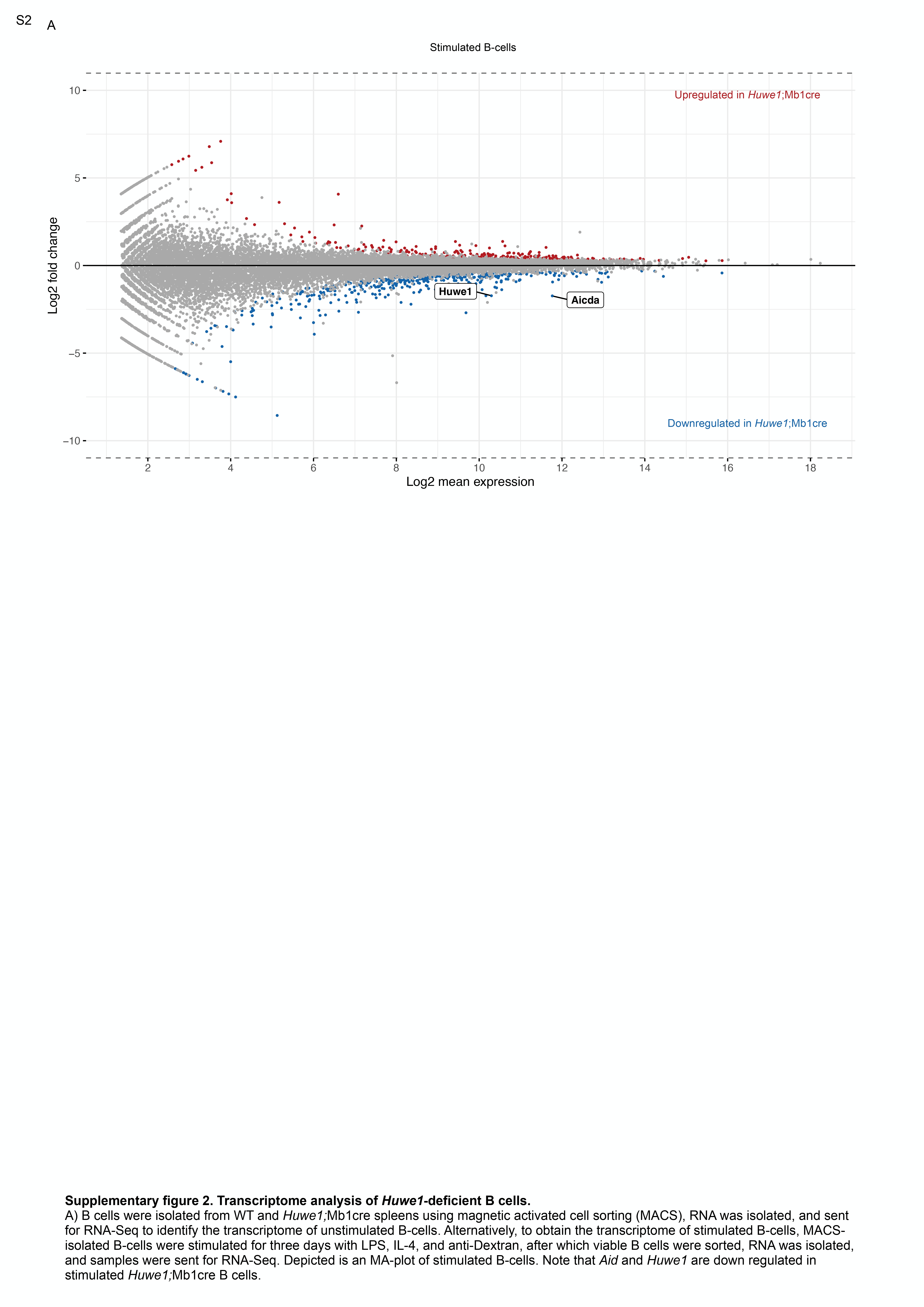

Supplement: Supplementary file 2 [file Image_2.jpeg]

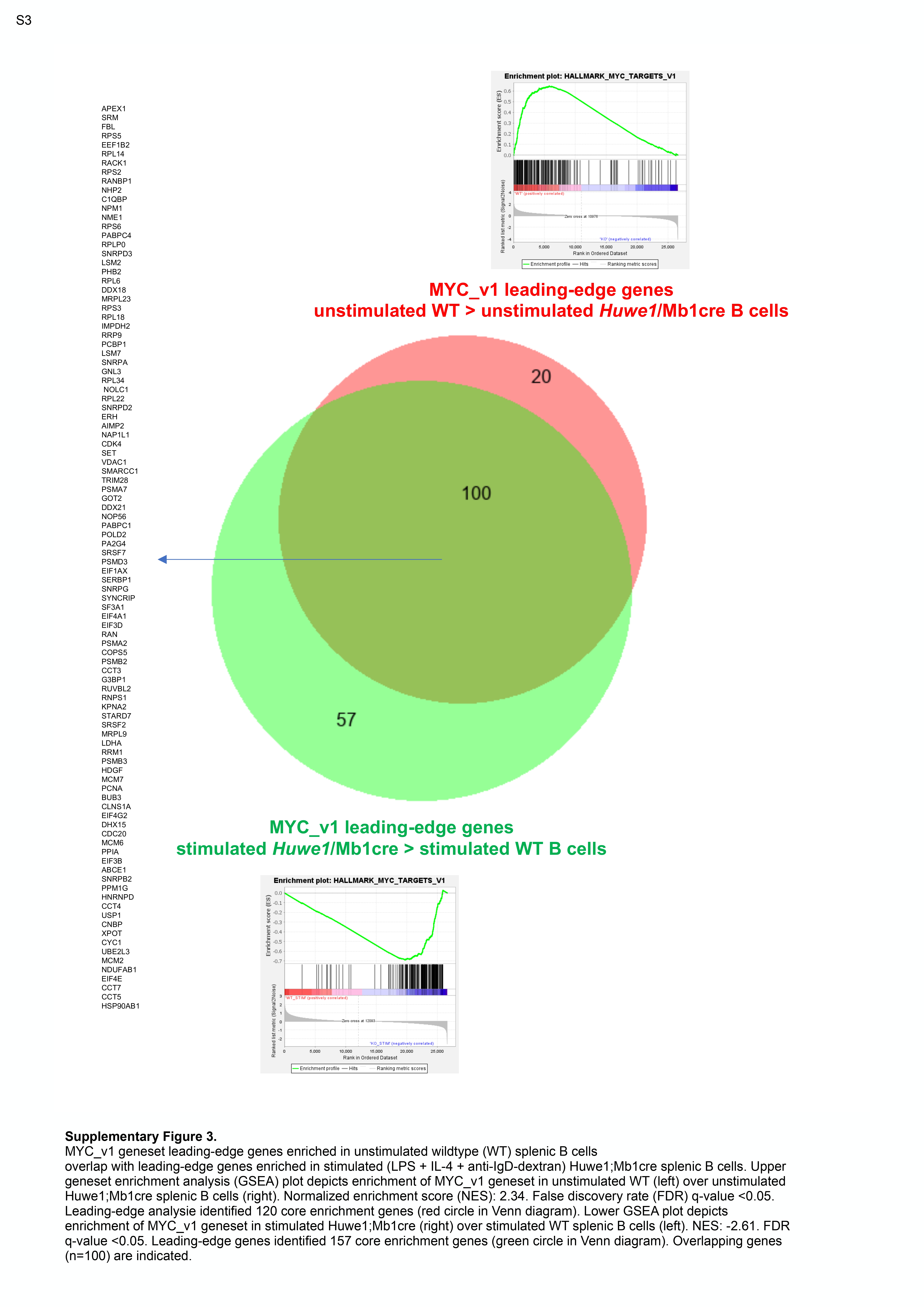

Supplement: Supplementary file 3 [file Image_3.jpeg]

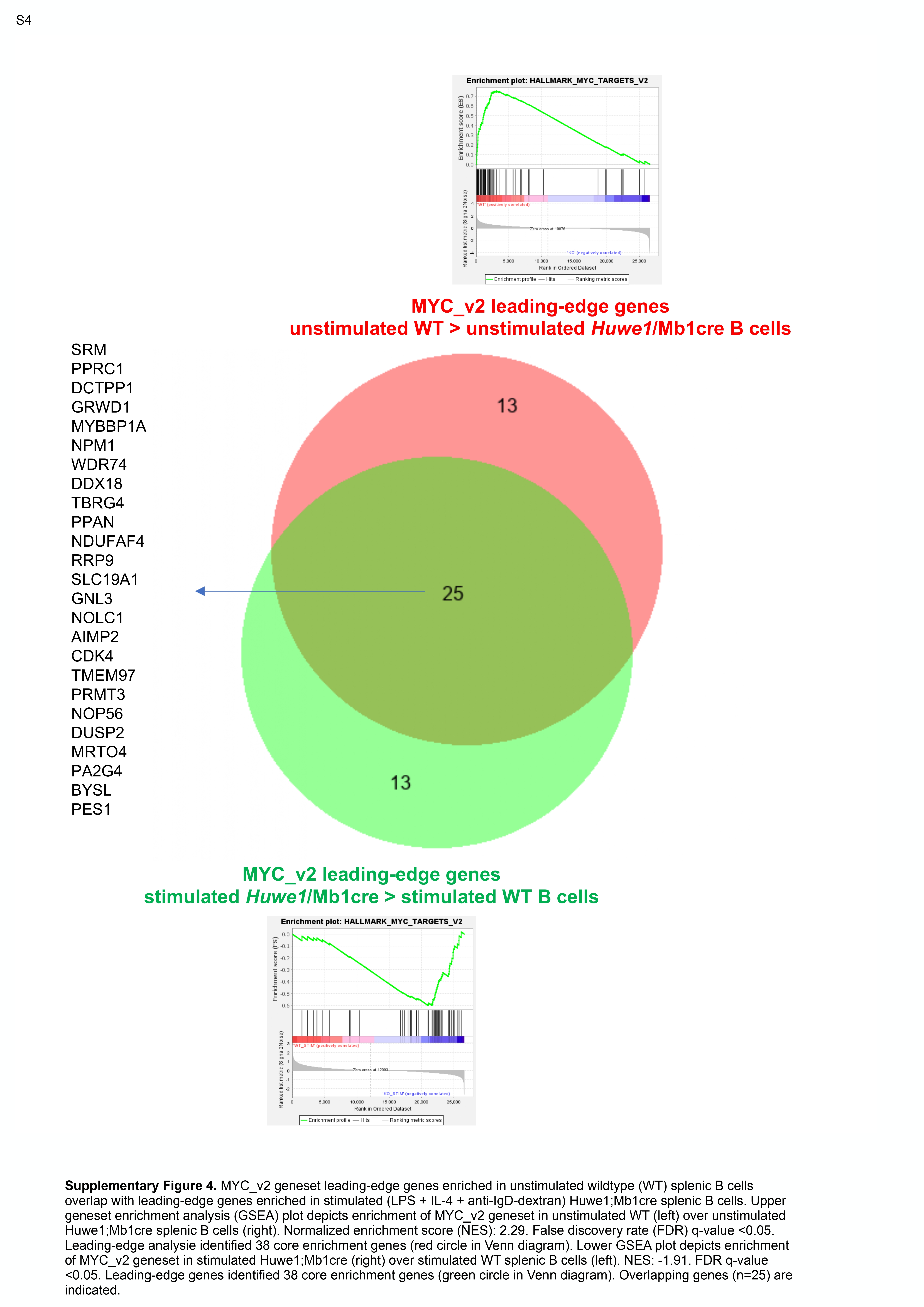

Supplement: Supplementary file 4 [file Image_4.jpeg]
