# Supplementary material for: Huwe1 supports B-cell development, B-cell-dependent immunity, somatic hypermutation and class switch recombination by regulating proliferation
Source: Front Immunol. 2023 Jan 9;13:986863. doi: 10.3389/fimmu.2022.986863 (PMC9869049; doi:10.3389/fimmu.2022.986863)
Supplement: Supplementary file 5 [file Image_5.pdf]

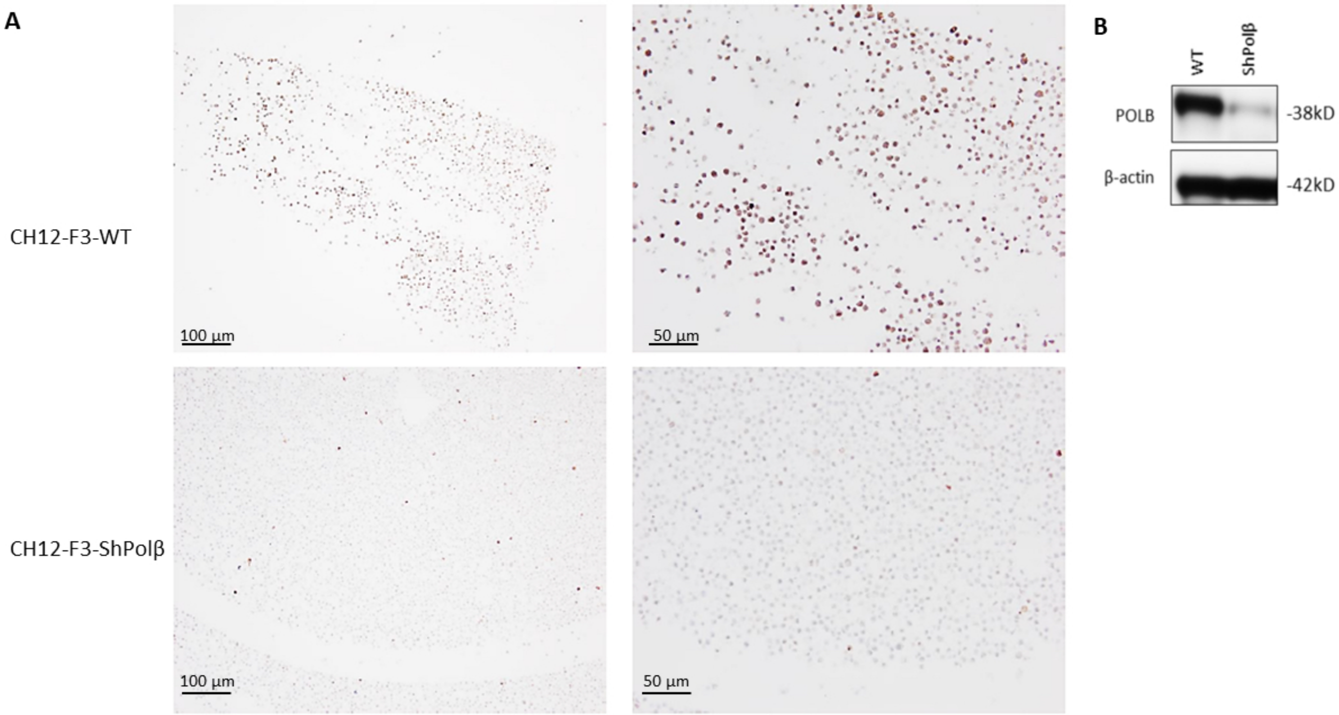

Supplementary figure 5. Validation of the specificity of the POLB antibody. To ensure that the ubiquitous POLB staining in splenic B cells is specific we used a POLB knockdown (shRNA) pool of the germinal center-derived mouse B-cell line CH12-F3 (CH12-F3-shPOLB). We validated the POLB antibody by immunohistochemistry (IHC) in paraffin-embedded samples, and by immunoblotting of protein lysates. The parental cell line (CH12-F3-WT) was used a positive control. (A) IHC staining for POLB visualized with 3'3-diaminobenzidine (DAB) in paraffin-embedded CH12-F3-WT (upper panel) and CH12-F3-shPOLB (bottom panel). Two different magnifications are shown. (B) Immunoblotting analysis of protein lysates from CH12-F3-WT and CH12-F3-shPOLB cells. For each lane, 50 micrograms of total protein was used. Beta-actin was used as a loading control.
